# Supplementary material for: Spatiotemporal orchestration of mitosis by cyclin-dependent kinase
Source: Nature. 2025 Jun 25;643(8074):1391–9. doi: 10.1038/s41586-025-09172-y (PMC12310531; doi:10.1038/s41586-025-09172-y)
Supplement: Supplementary file 2 — Reporting Summary [file 41586_2025_9172_MOESM2_ESM.pdf]

nature portfolio

Corresponding author(s): Nitin Kapadia

Last updated by author(s): May 13, 2025

Reporting Summary

Nature Portfolio wishes to improve the reproducibility of the work that we publish. This form provides structure for consistency and transparency in reporting. For further information on Nature Portfolio policies, see our Editorial Policies and the Editorial Policy Checklist.

Statistics

For all statistical analyses, confirm that the following items are present in the figure legend, table legend, main text, or Methods section.

n/a	Confirmed	
☐	☒	The exact sample size (*n*) for each experimental group/condition, given as a discrete number and unit of measurement
☐	☒	A statement on whether measurements were taken from distinct samples or whether the same sample was measured repeatedly
☐	☒	The statistical test(s) used AND whether they are one- or two-sided<br>*Only common tests should be described solely by name; describe more complex techniques in the Methods section.*
☒	☐	A description of all covariates tested
☒	☐	A description of any assumptions or corrections, such as tests of normality and adjustment for multiple comparisons
☐	☒	A full description of the statistical parameters including central tendency (e.g. means) or other basic estimates (e.g. regression coefficient) AND variation (e.g. standard deviation) or associated estimates of uncertainty (e.g. confidence intervals)
☐	☒	For null hypothesis testing, the test statistic (e.g. *F*, *t*, *r*) with confidence intervals, effect sizes, degrees of freedom and *P* value noted<br>*Give P values as exact values whenever suitable.*
☒	☐	For Bayesian analysis, information on the choice of priors and Markov chain Monte Carlo settings
☒	☐	For hierarchical and complex designs, identification of the appropriate level for tests and full reporting of outcomes
☒	☐	Estimates of effect sizes (e.g. Cohen's *d*, Pearson's *r*), indicating how they were calculated

Our web collection on statistics for biologists contains articles on many of the points above.

Software and code

Policy information about availability of computer code

Data collection	Microscopy images were collected using MicroManager (uManager, NIH) v2.0.0-beta3 20180704
Data analysis	Image analysis was done partly though FIJI 1.54f (NIH). Image and data analysis was also done using custom scripts with Matlab 2022. All custom scripts can be found at: https://github.com/nkapadia27/Spatiotemporal-Orchestration-of-Mitosis. DOI: 10.5281/zenodo.11072087

For manuscripts utilizing custom algorithms or software that are central to the research but not yet described in published literature, software must be made available to editors and reviewers. We strongly encourage code deposition in a community repository (e.g. GitHub). See the Nature Portfolio guidelines for submitting code & software for further information.

Data

Policy information about availability of data

All manuscripts must include a data availability statement. This statement should provide the following information, where applicable:

- Accession codes, unique identifiers, or web links for publicly available datasets
- A description of any restrictions on data availability
- For clinical datasets or third party data, please ensure that the statement adheres to our policy

All strains, data, and plasmids are available upon request. Due to the large size of the timelapse microscopy imaging data we have made this data available upon request by contacting the authors.

Research involving human participants, their data, or biological material

Policy information about studies with human participants or human data. See also policy information about sex, gender (identity/presentation), and sexual orientation and race, ethnicity and racism.

Reporting on sex and gender	Not relevant
Reporting on race, ethnicity, or other socially relevant groupings	not relevant
Population characteristics	not relevant
Recruitment	not relevant
Ethics oversight	not relevant

Note that full information on the approval of the study protocol must also be provided in the manuscript.

Field-specific reporting

Please select the one below that is the best fit for your research. If you are not sure, read the appropriate sections before making your selection.

☒ Life sciences ☐ Behavioural & social sciences ☐ Ecological, evolutionary & environmental sciences

For a reference copy of the document with all sections, see nature.com/documents/nr-reporting-summary-flat.pdf

Life sciences study design

All studies must disclose on these points even when the disclosure is negative.

Sample size	Sample sizes were not predetermined. For timelapse imaging, we aimed for at least 30-100 cells as our representative sample in our plots. We believe this was an adequate sample size as the phenotype and quantification (including distributions) was consistent across cells and across multiple repeats. For samples that were quantified, clear profiles in the distributions were obtained along with statistical results that gave us confidence in the conclusions we were making using the data. Furthermore, the parameter estimates, distributions, and statistics were consistent across repeats.
Data exclusions	Cells that did not properly grow or divide, including ones that led to cell death were excluded from analysis. Traces that were extremely noisy were removed after carefully looking at the raw data, as often these represented issues of image segmentation or poor quality of data. These excluded data often represented a very small fraction (1-5%).
Replication	All replicates were successful and we did at least 2 independent replicate experiments. For example, for timelapse imaging, we collected data for the same condition/strain at least on two different days to ensure consistency. Furthermore, we collected multiple fields of view during timelapse imaging.
Randomization	Randomization was not relevant to the study, as we only analyzed a single genotype for each experiment.
Blinding	Blinding was not relevant, as the phenotype of the cells was obvious when looking on the microscope. The analysis of the phosphoproteomics was based on grouping according to localization so it was not possible to perform this where the investigators were blinded to the groups allocation.

Reporting for specific materials, systems and methods

We require information from authors about some types of materials, experimental systems and methods used in many studies. Here, indicate whether each material, system or method listed is relevant to your study. If you are not sure if a list item applies to your research, read the appropriate section before selecting a response.

Materials & experimental systems

n/a	Involved in the study
☒	☐ Antibodies
☐	☒ Eukaryotic cell lines
☒	☐ Palaeontology and archaeology
☒	☐ Animals and other organisms
☒	☐ Clinical data
☒	☐ Dual use research of concern
☒	☐ Plants

Methods

n/a	Involved in the study
☒	☐ ChIP-seq
☒	☐ Flow cytometry
☒	☐ MRI-based neuroimaging

Eukaryotic cell lines

Policy information about cell lines and Sex and Gender in Research

Cell line source(s)	All S.pombe strains used are mentioned in Supplementary Table 1. The L972 was the background for all strains used in the experiments.
Authentication	Authentication was done by PCR and sequencing.
Mycoplasma contamination	Not possible
Commonly misidentified lines (See ICLAC register)	None

Plants

Seed stocks	*Report on the source of all seed stocks or other plant material used. If applicable, state the seed stock centre and catalogue number. If plant specimens were collected from the field, describe the collection location, date and sampling procedures.*
Novel plant genotypes	*Describe the methods by which all novel plant genotypes were produced. This includes those generated by transgenic approaches, gene editing, chemical/radiation-based mutagenesis and hybridization. For transgenic lines, describe the transformation method, the number of independent lines analyzed and the generation upon which experiments were performed. For gene-edited lines, describe the editor used, the endogenous sequence targeted for editing, the targeting guide RNA sequence (if applicable) and how the editor was applied.*
Authentication	*Describe any authentication procedures for each seed stock used or novel genotype generated. Describe any experiments used to assess the effect of a mutation and, where applicable, how potential secondary effects (e.g. second site T-DNA insertions, mosiacism, off-target gene editing) were examined.*
